# What Makes a Good Program? A Case Study of a School Admitting High Academic Achievers

**DOI:** 10.1100/tsw.2008.123

**Published:** 2008-10-10

**Authors:** Ching Man Lam

**Affiliations:** Social Welfare Practice and Research Centre, The Chinese University of Hong Kong, Hong Kong

**Keywords:** Project P.A.T.H.S., successful program, positive youth development

## Abstract

This paper reports the results of a qualitative study that explored the administration and implementation of the Tier 1 Program (Secondary 1 Curriculum) of the Project P.A.T.H.S. The case study method was used to explore perceptions of the teachers and the project coordinator of program effectiveness, and to identify various factors for program success. A school admitting high academic achievers was selected, and site visits, as well as individual and focus group interviews, were conducted with the program coordinator, social worker, and course teachers. The results suggested that clear vision and program goals, high quality of curriculum, helpful leadership, positive teacher attitude, and strong administrative support are factors for program success. Analyzing the data enables the researchers to understand the characteristics of a successful program as well as the interplay among factors for producing success.

### INTRODUCTION

The project is entitled "P.A.T.H.S. to Adulthood: A Jockey Club Youth Enhancement Scheme". The word "P.A.T.H.S." denotes **P**ositive **A**dolescent **T**raining through **H**olistic **S**ocial Programmes. The project is funded by The Hong Kong Jockey Club Charities Trust, and the research team is formed by academics of five Hong Kong universities, with The Chinese University of Hong Kong as the lead institution. This is a multiyear (2005–2009) universal positive youth development program, targeting adolescents studying in junior secondary schools (Secondary 1 to 3, or Grades 7 to 9). The purpose of the program is to promote the holistic development of young people by providing opportunities for them to learn competencies and skills that are conducive to positive development.

Based on the adolescent development issues observed in Hong Kong and the specific local context, a two-tier program was designed. The Tier 1 Program is a universal positive youth development program for students from Secondary 1 to 3. Each student will participate in either a 20-h full program or a 10-h core program in each year of junior secondary study. The Tier 2 Program is a selective program that targets adolescents with greater psychosocial needs.

Evaluation composed an important part of this project. Program evaluation is not a simple and straightforward task, and there are many types and approaches of evaluation[1,2,3]. For this project, both quantitative and qualitative evaluation strategies assessed program processes as well as the outcomes, and assessed effects of individual and multiple programs. While most of the evaluation data analyses are in process, the objective outcome evaluation[4], subjective outcome evaluation[5,6,7], the process evaluation findings generated from observation[8], and the interim evaluation[9] all support the effectiveness of the Tier 1 Program (Secondary 1 Curriculum). The focus now is precisely which factors make the program succeed. This paper reports social workers' and teachers' perceptions of the Tier 1 Program, and identifies factors that contribute to the program success, data that stem from a qualitative study that explored the administrative and implementation of the Tier 1 Program (Secondary 1 Curriculum). The case study method was used to explore perceptions (of the teachers, social workers, and project coordinators) of program effectiveness and to identify various factors for program success.

### BRIEF DESCRIPTIONS OF THE SCHOOL

The interviewed school is a high-band school in Hong Kong that admits high academic achievers. The banding system, which groups students according to their achievement levels, was adopted in 1978 as the procedure for allocating primary students to different secondary schools. Under this system, students are put under one of three different bands based on their achievement test scores in Chinese, Mathematics, and English near the end of primary school. Students with the highest achievement levels are allocated to the higher-band schools (i.e., Band 1) and those with lower achievement levels are placed in the lower-band schools (i.e., Band 3).

School environment also differs from band to band. In Band 1 schools, a majority of the children come from middle or high socioeconomic backgrounds, as most Band 1 schools are located in wealthier areas, such as Hong Kong Island and Kowloon. These schools often enjoy better facilities, have better teachers, and have more extracurricular programs than do low-band schools. Studies indicate that Band 1 schools advocate academic pursuits[10]. Teachers and parents tend to emphasize the importance of high achievement and set high standards for academic performance. Being branded as high-achieving learners, the students naturally have higher goals and higher expectations of themselves[11]. In addition, there is stronger parental commitment to education and the school culture values the quest for excellence.

Literature review reveals that most studies of high achievers focus either on the students' attributes/levels of competence[12,13] or their motivational goals[11], or compare the attributes of high achievers to low achievers[14] or gifted learners[15]. Current literature[11] describes high achievers as students who have high task and ego goals, and who report greater (when compared to low achievers) use of self-regulated learning strategies. They have been portrayed as possessing more positive than negative personality traits.

However, this encouraging picture is far from reality – the problems of high achievers have gone considerably unnoticed. Study results[16] indicate that high achievers are no less vulnerable to socially related self-concept problems than are members of the general population. Band 1 students in a highly competitive academic environment tend to focus on achievement through competing and comparing[17], and are more likely to face examination pressure or interpersonal-relationship problems. Academic issues[18], family problems[19], peer rejection[20], and personal distress[21] affect all adolescents, regardless of academic achievement. The common saying, "Band 1 students have no problems" is a misconception and is far from the truth.

The Project P.A.T.H.S., for its part, considers adolescent development in a holistic manner. The program is particularly fit for students of Band 1 schools, as it focuses not only on academic excellence, but also on cognitive, emotional, social, and spiritual aspects, achieving the goal of building up the developmental assets of adolescents.

The participating school has characteristics of a typical Band 1 school. It is located in a prosperous area of the Kowloon peninsula. Nearly 80% of the students come from middle-class families with middle to high socioeconomic backgrounds. Most are the only child and their families are able to afford domestic helpers to take care of their everyday needs. The school culture promotes the pursuit of academic excellence and helps students to develop in different aspects. The students are exposed to various opportunities and can participate in a wide range of extracurricular or skill-training activities, either provided by the school or arranged by the parents.

The school started to participate in the Project P.A.T.H.S. in the academic year 2006/07. The school had 200 Secondary 1 students, divided into five classes, each with a class teacher. For launching the Tier 1 Program (Secondary 1 Curriculum), the school adopted the 20-h full program (two 2.5-h sessions, one 2-h session, and 13 1-h sessions), which was broken down into 16 sessions during the class teachers' periods, 10 sessions of Life Education, and three activity days.

The three activity days were scheduled at the beginning and end of the program. Before implementation, introductory sessions were arranged on the Secondary 1 orientation day – one session for parents and one for students. In order to gain parental support and to familiarize parents and students with the program, the P.A.T.H.S. team briefed them on program rationales and format. The conclusion session, held during the postexamination period, included a closing ceremony and program summary.

With regard to manpower, there were six teachers, one school social worker (stationed in the school), and five agency social workers involved in conducting the Tier 1 Program. There were eight teachers, one school social worker, and three agency social workers who participated in the preimplementation training workshops arranged for the instructors conducted by the P.A.T.H.S. project team. The Tier 1 Program was mainly taught by class teachers, with the agency social worker assisting. The school used Chinese as the medium of instruction for the program.

Before launching the program, the school arranged about seven to eight lessons for collective preparation. The team first reviewed the manual to get familiar with the curriculum, then discussed, and then selected (with frequent compromises) the most suitable curriculum components as teaching materials. Teachers took turns preparing for the sessions. The responsible teacher would plan for the session, prepare teaching resources, make copies for notes, and prepare program materials and prizes for other colleagues.

Among the six teachers involved in this project, one was the program coordinator. Each of the other five teachers was responsible for one class – his/her role was teaching the curriculum, monitoring classroom discipline, preparing teaching materials, and coordinating program-related matters. The social worker's role was to coteach the program, prepare teaching materials, and coordinate program-related matters. In addition, the social worker networked five social workers from the Youth Integrated Service Team of a local youth agency to assist in teaching three half-day Tier 1 special lessons and to help with the Tier 2 Program.

The program coordinator and the school social worker monitored the progress of the curriculum and helped in group discussion. The coordinator was responsible for overall coordination, administrative work support, and lesson arrangements. The school social worker played a support role and was responsible for networking and resources support role. The class teachers, with the assistance of the social workers, were responsible for teaching and program implementation. Upon the completion of the Tier 1 Program, there was a final evaluation meeting, where the instructors evaluated the program and made suggestions for Secondary 2 curriculum.

### METHODS

#### Participants and Procedures

In academic year 2006/07, a total of 207 schools joined the Project P.A.T.H.S. (Secondary 1 Curriculum). The school in this study was randomly chosen and invited. A member of the P.A.T.H.S. training team (who supported this school all through the preparation and implementation process) conducted site visits to the school. The author, a P.A.T.H.S. research team member, and this training team member, a trained social worker with a Master degree in Social Work, conducted the interviews. Interviews with the school coordinator and school social worker, and a focus group interview with the program coordinator, five instructors, and one school social worker, were conducted separately. Prior consent from the school principal was obtained for the school's participation, as was informed consent from the respondents, who knew the purpose of the interview and who were informed of the principle of confidentiality. The interview with the school coordinator and social worker took 50 min, while the focus group interview lasted 80 min. The interviews were conducted in mid-June 2007, after the school had completed the Tier 1 Program.

### Instruments

A specific, self-constructed, semi-structured interview guide was designed for the interviews. The interviewing guide was divided into three parts:

- Respondents' perceptions of the school's administrative arrangement, such as preparation and support from within the school and from the social work agency
- Respondents' perceptions of the program implementation process, such as teaching process and techniques, students' worksheets, and student assessment
- Respondents' overall perceptions of program effectiveness and program success

The school was also required to complete a background information questionnaire. All data collected from the questionnaires were typed by student helpers; the recordings of the interviews were transcribed with Chinese word-processing software and checked by the second interviewer.

The qualitative data analysis[22,23,24] had two steps. First, words, phrases, and/or sentences that formed meaningful units at the raw response level were coded. Second, the codes were further combined into broader themes. The qualitative data were coded and categorized by the research assistants and further checked by the interviewers. The study met the principles of qualitative analysis[25]: data were double checked to ensure trustworthiness, triangulation by researchers ensured that the inquiry process was without bias, raw and categorized data were kept in a systematic filing system to ensure that the findings were auditable, and the researchers were conscious of their own biases for/against, preoccupations with, and expectations of the program.

## RESULTS

### Perception of Administrative Arrangement

Good administrative management was repeatedly mentioned in individual and focus group interviews as playing a significant role in program success at the participating school. The administrative arrangements included consultation with teachers before program implementation, lesson schedules, shared workloads among teachers, leadership by the program coordinator, and expectations of selected teachers.

Before the implementation of the Project P.A.T.H.S., the coordinator introduced the project to the staff in different staff meetings. After deciding to join, all colleagues in the school utilized consultations in staff meetings to get ready for the program. The coordinator indicated how the school prepared for the project.

> *"After we attended the briefing session (by P.A.T.H.S. research team) in 2005, we introduced the project in the staff meeting several times. We decided that we would join this project in 2006 and announced that we would approach individual teacher to seek their assistance. Although we didn't know who would be the ones involved in the program, we all prepared for the program. Finally, we picked five colleagues. For the selected teachers, of course, it*

> *was a pleasant surprise (you jing you xi). Then we decided that the teachers, the social worker and I should all go for training, and we joined the training workshop together."* (Individual interview with program coordinator)

The narrative reflected another advantage of early preparation – the school could "provide early notification to teachers responsible for teaching the program. Then, they can do early preparation and go for the training." Because of early preparation, there were eight teachers, one school social worker, and three social workers from the local Youth Integrated Service Team assisting in the Project P.A.T.H.S. of this school who had participated in training workshops conducted by the Project P.A.T.H.S. research team. With sufficient training and preparation, the program started smoothly.

Making good lesson arrangements, recognizing the workload of colleagues, and sharing the workload among teachers were also good administrative strategies perceived by interviewees. With regard to lesson arrangements, the school integrated the program into existing school lessons with similar content and topics. School assemblies, class teacher lessons, Life Education lessons, and existing special activities such as Secondary 1 orientation day were lessons used to incorporate the P.A.T.H.S. curriculum. This arrangement avoided giving teachers extra work, and students valued the learning as part of the normal curriculum. As Mr. A (anonymity was used according to order of response during interviews), the program coordinator, said about his feelings toward the workload recognition: "In the past, all I need to do is just attend the assembly, but now I need to teach. So, when the Principal announced that we can count this as one lesson (i.e., one teaching hour), we feel much better – this is much fairer."

The work was divided among teachers through joint effort and mutual support. Their team divided "two to three sessions as a unit", and the teachers conducted "collective preparation meetings" to decide the suitability of curriculum. During the meetings, the responsible teacher presented the teaching plan for comment. Finally, there would be "an agreed teaching plan" and "collective preparation". Instead of printing worksheets or purchasing prizes individually, materials were prepared centrally for all classes. This arrangement helped to share the workload and avoided duplication.

Good leadership is an important factor that the interviewees also cited for program success. According to the teachers, the program coordinator provided ongoing support. He was the first one to attend the training course, and he shared the program rationales with other team members, arranged team meetings for teachers to prepare the course together, and gave good administrative support such as lesson rearrangement. During program implementation, he went to classrooms to understand what was happening and provide immediate help. As Mr. B said: "I saw that Mr. A was so committed, and it made me try my best. So the 'Chief Chef' is very important. His role is to organize us, providing a bridge to the social worker…I was unable to attend the camp activity because it clashed with my studies. Mr. A took up my part. Not only me – when other colleagues had time conflicts, we have mutual support and mutual tolerance…" In the eyes of the colleagues, the program coordinator demonstrated leadership and helped build a supportive team spirit.

Last, but definitely not least, another good administrative arrangement was manpower deployment. According to the program coordinator, the principal selected teachers who were "responsible", "committed", and "with mission" to implement the Project P.A.T.H.S. In fact, colleagues also said the principal "selected colleagues with 'heart' for this program" and showed support for this project. Teachers who taught P.A.T.H.S. had some of their workload released or assumed by other teachers. The participants appreciated the manpower deployment policy and it facilitated the implementation of the program.

### Perception and Feelings of Teachers about the Program

The teachers perceived the program as having a clear goal, and they supported the program and its rationales. They regarded the program as successful, saying, "The P.A.T.H.S. program was more comprehensive than the existing programs of similar nature." Said Mr. B in the focus group interview, "I am sure that the students need a course of this kind." Ms. C agreed with the program objectives and regarded the curriculum content as helpful to students. Mr. B shared a similar opinion. He saw the curriculum as "well designed" and the teaching materials as "helpful". It helped to "save our time for preparation" and "all we needed to do was to make some minor modifications to fit the needs of the students".

With regard to program effectiveness, the program coordinator, teachers, and social worker all pointed to the positive impact of the program on students. Mr. B clearly affirmed this, saying, "Although it is difficult for students to understand the program rationales, they can still learn a lot…Their emotional control and family relationships have been improved," and "The course improves students' behavior, helps students build confidence and strengthens classmate relationships."

Another positive effect of the program was enhanced teacher-student relationships. As stated by Mr. A, the program coordinator, and Mr. B, "The relationships between teachers and students are getting closer. We grow together…besides, the curriculum content touch on family and interpersonal relationship, (which helps) enhance our mutual understanding."

Ms. C echoed this opinion, describing the program's effects as "sharing and more mutual understanding in class". School life was "happier and there (were) fewer students breaking the rules and regulations". The program gave the students "a chance to speak out". She said, "We can't ensure that they will follow the rules in the future, but at least they know they should respect people, respect life and obey the rules. In their daily conversation, you can observe that students mention the terms learned in the project. At least they know."

The course coordinator and teachers involved were also very positive about the program:

> *"If I had to mark the Tier 1 Program, I would give 80 marks because I know that students have benefited. Our school also benefits – we got additional resources like teaching kits. Students like these lessons, and they give positive feedback. At least 70–80% of the students said they like the classes."* (Mr. A, course coordinator, individual interview)

> *"For students, this program is successful. If marking, it's…80"* (Mr. B, focus group)

Mr. A, the program coordinator, could see the long-term effects:

> *"In the long run, after we run the program for three years, the curriculum has become an integrative part in our 'class teacher lessons' (ban zhu ren ke). Teachers have training (for this kind of program). After three years, we have a group of teachers who have experienced teaching the growth and personal development course. We hope that the curriculum can be extended to Forms Four, Five and Six, so that all forms will have courses of this nature…"*

Data from focus groups gave evidence of program effectiveness – students were able to remember the course content, had better emotional control, respected life and themselves, were more willing to obey the school rules, and had better behavior, confidence, and relationships with classmates. According to Mr. B, "Overall speaking, the school life is happier…(the program) benefits not only the student – we benefit, too. For me, it is not only teaching the students, but also myself."

### Perception of Support from the P.A.T.H.S. Team

As revealed in the interviews, there were several aspects of support from the P.A.T.H.S. research team and from the social work agency during implementation. First, the P.A.T.H.S. research team provided a 3-day training course for teachers and social workers involved in the program. All the interviewees revealed that the training course was helpful in preparing them for the program. As one teacher, Ms. D, said in the focus group, "The workshop leads to self-growth and self-awareness. I understand my thoughts and feelings through participating in the workshop." According to the participants, the training workshop went beyond the skill-training level to reflect on the personhood of the participants. It helped participants to rethink and reflect, achieving the goal of professional and personal development.

Second, the P.A.T.H.S. research team organized sharing sessions for the participating schools, deeply appreciated by participants. In order to develop a mutual support network and to provide chances for colleagues from different schools to learn from each other, sharing sessions were arranged from time to time and colleagues were invited to share their experiences. The opportunity for interschool sharing, the teachers said, “made us not feel so lonely”. After learning the experiences from other schools, the teachers knew “that we are not alone”.

Third, the participants perceived the “Cowalker” Scheme as a strong source of support. The P.A.T.H.S. research team arranged a colleague to be a “cowalker” for each participating school. His/her role was to pay regular visits to the school, provide consultation to teachers and social workers, and support the school throughout the process. According to the interviewees, the staff from The Chinese University of Hong Kong as “cowalkers” made them feel “good” and “taken care of”. The interviewees were satisfied with and appreciated the “comprehensive training” and “comprehensive support” provided by this project.

Social work agencies were another source of support and satisfactory collaboration. The social worker stationed in the school networked five social workers from the Integrated Youth Service Team in the district to support the project. According to the teachers, partnering with the district social work agency “reduces the burden and workload” and “helps lead the class”. The agency’s help was particularly helpful for the three combined classes. With the help of the social workers, the teachers could introduce “game elements” to make the program content more “lively”. Besides, all five agency social workers had gone through the training workshop in the early summer. They “shared the program goals” and discussed “how to share workload and how to arrange for division of labor in the late summer”.

#### Perceived Difficulties

Other than the success factors, difficulties and constraints were also mentioned. Some students said they had learned the curriculum content in primary school. Another constraint frequently mentioned by teachers was class size and the in-depth curriculum. With about 40 students and rich program content, there was insufficient time for class discussion. Of all the limitations, people factors were the foremost concern. The program coordinator and the interviewees revealed that it was difficult to find “suitable people to teach the program”, and they worried that when the program came to full implementation, they would not be able to get enough “suitable” teachers.

### DISCUSSION

There are several interesting points about what makes a good program. The accounts of the interviewees make it very clear that program goals and rationales, program leader, staffing, and administrative support are major factors. These findings are consistent with Anderson and Pellicer’s[26] conceptual framework for a successful program, which included four interrelated constructs: (1) program effectiveness standards, referring to clear purposes and high-performance standards; (2) school culture, referring to strong community support and talented, hardworking teachers; (3) curriculum, referring to opportunities to learn and curriculum integration; and (4) teaching, referring to concern for students and nonacceptance of failure. The research findings are also consistent with other school research, which addresses the importance of leadership[27,28] and supports the importance of teacher quality[29,30,31].

The case study results suggest that if teachers concur with the program’s goals and rationales, they will see the importance of the program and try their very best to promote the program’s message and themes. In a Band 1 school, with much emphasis on academic excellence, the program is able to involve teachers who identify with the ideology of holistic adolescent development, and who are willing to focus more on task and mastery rather than competition; these are factors for program success. A program that is well designed, with clear goals that match adolescent needs, and that teaches not only theory, but also practical knowledge and skills, can be a successful program.

Despite the fact that Band 1 students demonstrate better academic performance than students of other bands, they still have a need to cultivate psychosocial competencies. The problems of underachievers draw much attention, but the needs of high achievers have been ignored. Literature review reveals that a majority of the studies on high achievers have focused on their attributes or levels of competence[12,13], with very little literature on their needs or the crises that they are likely to encounter. School principals, teachers, parents, social workers, scholars, and the general public have the misconception that Band 1 students have fewer or even no problems. However, newspaper reports show that students from high-band schools continue to engage in criminal behaviors, such as thieving, paparazzo, or posting obscene magazine photos on the Internet. Studies reveal that high achievers pay a price for their success[32]. They have feelings of emptiness and are stressed by the pursuit of success. The program helps students of Band 1 schools by providing opportunities for students to develop bonding relationships, promoting social competence of students, cultivating their resilience, and providing opportunities for prosocial involvement. Students are able to experience well-rounded development through the program.

The study also reveals that even in a Band 1 school, which places great emphasis on academic achievement, a program with clear goals that match the parents' needs, the students' needs, and also the context of learning can be a successful one. High-achieving students have a desire to learn[11]. If a program meets their needs, they will have motivation to learn. The P.A.T.H.S. program in this school has teachers who concur with the program goals and convey those goals to parents and students through orientation sessions before program implementation. With parental support and endorsement, and student appreciation, the program runs smoothly.

Administratively, the school needs to fit the program into the school policy and provide administrative support for teachers. Allowing sufficient time for preparation, training teachers before they begin teaching, giving teachers the choice of whether to join the project, making lessons part of the school curriculum, using good manpower deployment strategies to form a supportive team, and getting external support from the P.A.T.H.S. research team and social work agency – all these help to facilitate program implementation.

Lastly, and perhaps most significantly, is the "people" factor. Undoubtedly, the presence of a responsible program coordinator and self-motivated teachers is the most important facilitating factor. When conducting the case study, we observed a dedicated team with shared leadership, good division of labor, and mutual support among colleagues. During the course of the interviews, the teachers emphasized not only academic pursuits, but also the students' growth and development. They also stressed their personal growth and professional development. The study results inform us that, as program designers, we need to be concerned not only for the needs of students, but also for the needs of the instructors. The reality is: Dedicated teachers have a higher risk of burnout. Our challenge is: How can their enthusiasm and devotion be maintained? The positive youth development program should also be a positive *teacher*-development program.

### ACKNOWLEDGMENTS

The preparation for this paper and Project P.A.T.H.S. were financially supported by The Hong Kong Jockey Club Charities Trust.
